# Fatigue after pulmonary embolism—prevalence and predictors

**DOI:** 10.3389/fcvm.2026.1882131

**Published:** 2026-08-24

**Authors:** Marie Schmitz, Simone Fischer, Thomas M. Berghaus, Markus Naumann, Jakob Linseisen, Christine Meisinger, Inge Kirchberger

**Affiliations:** 1 Epidemiology, Medical Faculty, University of Augsburg, Augsburg, Germany; 2 Institute for Medical Information Processing, Biometry, and Epidemiology—IBE, LMU Munich, Munich, Germany; 3 Pettenkofer School of Public Health, Munich, Germany; 4 Department of Cardiology, Respiratory Medicine and Intensive Care, University Hospital Augsburg, Augsburg, Germany; 5 Medical Faculty, LMU Munich, Munich, Germany; 6 Department of Neurology and Clinical Neurophysiology, University Hospital Augsburg, Augsburg, Germany

**Keywords:** cardiovascular disease, comorbidities, depression, fatigue, post-PE syndrome, pulmonary embolism, quality of life

## Abstract

**Introduction:**

Pulmonary embolism (PE) is a common condition with negative long-term consequences such as reduced quality of life. However, there is little knowledge regarding fatigue after PE. The aim of this study was to determine prevalence and predictors of fatigue after PE.

**Methods:**

The Lungenembolie Augsburg (LEA) study includes patients treated for acute PE at the University Hospital of Augsburg, Germany. Patients were interviewed during hospitalization and received postal questionnaires after 3 and 12 months. Fatigue was assessed using the Fatigue Assessment Scale (FAS). Data from the “Stroke Cohort Augsburg (SCHANA)” study and the “Metabolism, Nutrition and Immune System in Augsburg (MEIA)” study were used to compare fatigue prevalence after PE with that after stroke and in the general population. Multiple linear regression models were built to identify baseline predictors for fatigue 3 and 12 months after PE.

**Results:**

Three months after PE, the age-sex-standardized fatigue prevalence was 40.76% [95% confidence interval (CI): 24.81%–56.71%] and 12 months after PE it was 32.36% (95% CI: 20.72%–44.01%), which was significantly higher than in the general population (12.68%; 95% CI: 9.75%–15.61%), but did not significantly differ from the prevalences found in patients with stroke (3 months: 30.97% (95% CI: 22.73%–39.22%); 12 months: 28.02% (95% CI: 17.49%–38.55%). Multiple linear regression models revealed that gender modified the relationship between depression at baseline and fatigue 3 and 12 months after PE. In men, Hospital Anxiety and Depression Scale (HADS) Depression score (*β* = 1.92, *p* < 0.0001) as well as school education >9 years (*β* = −3.72, *p* = 0.0491) and in women, HADS Depression score (*β* = 0.80, *p* = 0.0057), were significantly associated with fatigue 3 months after PE. Twelve months after PE, HADS Depression score (*β* = 1.69, *p* < 0.0001) and school education >9 years (*β* = −5.28, *p* = 0.0059) were significantly associated with fatigue in men.

**Discussion:**

The results show that fatigue is common after PE. Early screening for depression and fatigue in both men and women should be considered. Lower school education level in men was shown to be a predictor of fatigue and should be taken into account when screening patients.

## 1 Introduction

Pulmonary embolism (PE) is a common and often fatal condition, with an estimated incidence of 50–100 cases per 100,000 person-years. It ranks as the third most frequent cardiovascular disease after myocardial infarction and stroke and is fatal in 7%–11% of cases (1). The risk of developing PE increases with age, and with the aging population in Western societies, the incidence of PE is expected to rise further (1). Meanwhile, early mortality after PE is decreasing (2), resulting in a growing number of people living with the long-term consequences of the disease. As a result, the management of long-term effects is becoming an important component of patient care.

Studies show that up to one half of the patients with post-acute PE suffer from long-term negative effects such as dyspnea, reduced quality of life and functional limitations, which are collectively referred to as post-PE syndrome (3). A significant predictor of mental and physical quality of life in patients with venous thromboembolism, which includes PE, is fatigue (4). Fatigue is characterized by a persistent lack of energy that cannot be compensated by typical energy-restoring strategies and limits patients' ability to carry out everyday activities (5). Fatigue can manifest psychologically (cognitive and emotional symptoms) and physically (loss of energy, weakness, sleepiness) (6). Having severe fatigue with exhaustion has also been identified as risk factor for stroke in women and coronary heart disease (5, 7, 8). In addition, fatigue is associated with increased health care utilization and incapacity for work (9).

Fatigue—specifically brief periods of fatigue related to situational factors such as lack of sleep or stress—can be observed in the general population (10). However, longer lasting and more intense forms of fatigue are often associated with chronic somatic diseases such as Parkinson's disease, multiple sclerosis, congestive heart failure and stroke (5, 10). The etiology and pathophysiology of fatigue remain poorly understood, which complicates both diagnosis and finding of effective therapeutic interventions (11).

Although fatigue has been described as part of the post-PE syndrome, there is still very limited knowledge regarding fatigue following PE (3). Fatigue and loss of energy were described as the two most common negative side effects in a qualitative study with 16 patients 6–12 months after PE (3). Patients reported different experiences, some described an increased need for rest and sleep, while others mentioned reduced strength during physical activity (3). In a large retrospective analysis of a US insurance claims database, new fatigue or malaise 3–12 months after PE was found in 18.5% of patients (12). A comparison between patients with deep vein thrombosis and PE showed worse results for mental fatigue in patients with PE (4). A link between PE and fatigue has also been found in cancer patients (13).

Despite increasing attention to the long-term effects of acute PE, the prevalence and predictors of fatigue as a distinct symptom remain poorly understood. Therefore, the aim of this study is to determine the prevalence and identify predictors of fatigue after PE.

## 2 Materials and methods

### 2.1 LEA analysis sample

The present study used data from the “Lungenembolie Augsburg Studie (LEA)”. The LEA study is a cohort study that includes adults with confirmed acute PE who have been admitted to the University Hospital of Augsburg, Germany. Between 1 July 2017 and 1 July 2023, 977 patients were included in the study. Patients were followed up after 3, 6 and 12 months and after that yearly up to 5 years (1). The study was performed according to the Declaration of Helsinki. Ethical approval for the study protocol was obtained from the Ethics Committee of the Ludwig-Maximilians-University Munich (Date of approval: 1 August 2017, Reference number: 17-378). In our analysis, we included patients who participated in the baseline assessment as well as the 3-month and/or the 12-month follow-up and who filled out the Fatigue Assessment Scale (FAS) at the 3-month and/or the 12-month follow-up. The FAS was implemented in the study starting from June 2021.

### 2.2 Data collection

The baseline assessment included standardized interviews carried out by study nurses during the hospital stay. The interview covered socio-demographic variables, comorbidities, symptoms on presentation, delay in diagnosis, and risk-factors for PE (1). The patients also completed self-reported questionnaires (see Table 1). Fatigue was assessed using the Fatigue Assessment Scale (FAS), which evaluates both mental and physical fatigue and has been used to assess fatigue in a variety of conditions, such as chronic heart failure, stroke and cancer (7, 14). The FAS has demonstrated high reliability and validity when used in the general population and is a valid tool for measuring fatigue in stroke patients (15, 16). Since there is no literature on using the FAS in PE, we applied the cut-off value identified as optimal for classifying post-stroke fatigue (FAS score ≥ 24), as they are both cardiovascular diseases (17).

**Table 1:** Characteristics and implementation of self-reported questionnaires used in the “Lungenembolie Augsburg (LEA)” study.

Questionnaire	Baseline	3-Month follow-up	12-Month follow-up	Number of items	Range	Interpretation	References
Fatigue assessment scale (FAS)		X	X	10	10–50	<24 = no fatigue ≥ 24 = fatigue	(17, 18)
Hospital anxiety and depression scale (HADS)—subscale depression	X	X	X	7	0–21	<11 = no depression ≥ 11 = symptoms likely correspond with a clinical diagnosis of depression	(19, 20)
European quality of life questionnaire visual analog scale (EQ-5D-5L VAS)	X	X	X	1	0–100	Higher score = better overall perceived health status	(20)

Clinical data was collected during the hospital stay including information on medication, diagnostic procedures, comorbidities, risk factors for PE, and disease severity (1). We used the patient's medical chart for information on pre-existing conditions. If this information was missing, it was taken from the patient's interview. The simplified PESI score (sPESI score) was calculated. This score includes information on age, comorbidities, oxygen saturation, heart rate and blood pressure and is used to predict the mortality risk after acute PE, with a score ≥ 1 indicating an elevated risk of death in the first 30 days after the acute event (18).

### 2.3 Comparison samples

To compare the fatigue prevalence after PE to the fatigue prevalence after stroke and in the general population, data from two other studies, which also applied the FAS as a measure of fatigue, were used. First, we used data from the “Stroke Cohort Augsburg (SCHANA)” study. This observational cohort study included adult patients (≥18 years) treated for stroke at the University Hospital of Augsburg, Germany (21). Between September 2018 and November 2019, 945 patients were included in the study (14). The study was performed according to the Declaration of Helsinki. The study protocol was approved by the Ethics Committee of the Ludwig-Maximilians-University Munich (Date of approval: 29 May 2018, Reference number: 18-196). Patients who filled out the FAS at the 3-month follow-up and/or the 12-month follow-up were included in the present analysis sample.

Second, we used data from the cross-sectional “Metabolism, Nutrition and Immune System (MEIA)” study (22). This study was carried out between 2021 and 2023 and included data from 594 randomly selected inhabitants of the Augsburg region aged 18–75 years (22). Ethical approval was obtained from the Ethics Committee of the Ludwig-Maximilians-University Munich (Date of approval: 13 July 2020, Reference number: 20.334). The study was performed according to the Declaration of Helsinki. Participants who filled out the FAS were included in the present analysis.

### 2.4 Statistical analysis

Frequencies with percentage values and median with interquartile range (IQR) were reported for socio-demographic and health-related characteristics of the LEA sample at baseline. For the 3-month and the 12-month follow-up, the same characteristics were reported stratified by FAS score ≥ 24 and FAS score < 24. Mann–Whitney-*U*-Test, Chi-Square-Test and Fisher's Exact Test were used to compare characteristics of patients with fatigue and without fatigue. Characteristics of the SCHANA sample and the MEIA sample were reported by frequencies with percentage values and median with interquartile range (IQR).

Age-sex-standardized fatigue prevalence was obtained from the LEA 3- and 12-month follow-up samples, the SCHANA 3- and 12-month follow-up samples and the MEIA sample. We analyzed the age distribution by categorizing the samples into age strata from 19 to 75 + years (19–24, 25–29, 30–34, …, 75+) and calculated age-sex-standardized prevalences with direct standardization using the European Standard Population 2013 stratified by gender (23). Age-sex-standardized prevalence was compared between the samples using Chi-Square-Test.

Multiple linear regression models were built to investigate the association between baseline predictors and fatigue at 3 and 12 months after PE. The choice of possible predictors was based on literature. Since there is no literature on predictors of fatigue after PE yet, potential predictors were selected based on studies of other cardiovascular diseases such as stroke and myocardial infarction. Age and gender (24–26), social support (24, 27–29), education (27), smoking (30), diabetes (25, 26), and depression (14, 24, 25, 27–29, 31, 32) were reported to be predictors of fatigue. sPESI score was included in the regression models because some studies found an association between severity of disease and development of fatigue (14, 24). Finally, since it has been shown that COVID-19 is associated with an increased risk of fatigue (33, 34) we also included a history of COVID-19 as covariable.

Due to a significant interaction between gender and HADS Depression score, models were stratified by gender. Model performance was assessed using Adjusted *R*^2^ and Akaike Information Criterion. Assumptions of multiple linear regression were tested as follows: Linearity with polynomials, normal distribution of residuals with residual plots in combination with Shapiro–Wilk-Test, homoscedasticity with residual plots in combination with Breusch-Pagan-Test, outliers with Cook's distance and multicollinearity with variance inflation factors (VIF).

Sensitivity analyses were conducted to assess the influence of outliers on the model by calculating robust multiple linear regression models and comparing the results with the multiple linear regression models. Robust regression was performed via M-estimation, which assigns weights close to one to observations with small residuals and weights close to zero to observations with large residuals (35). The model therefore provides more robust parameter estimates by reducing the influence of outliers (35). Furthermore, we tested the robustness of the model by removing the variable HADS Depression score. Additionally, we compared baseline characteristics of the analysis sample to patients who dropped out after baseline assessment. All analyses used an alpha-level of 0.05 and were performed using R version 4.3.3.

## 3 Results

### 3.1 Sample characteristics

Out of 977 patients included in the LEA study, 811 had a baseline interview and 556 participated in the 3-month and/or the 12-month follow-up. Since the FAS was not administered in the first 4 years of the study, only a small proportion of the total sample was able to complete the questionnaire (205 patients at the 3-month and/or the 12-month follow-up). The 3-month follow-up analysis included 132 patients, while the 12-month follow-up analysis was comprised of 148 patients who completed the FAS. Baseline characteristics of the included patients can be found in Table 2. The analysis sample consisted of 113 men (55.12%) and 92 women (44.88%) with a median age of 63.00 years (IQR: 55.00–74.00) at time of PE. Patients' medical history showed that hypertension was the most frequent diagnosis in 49.76%, followed by cancer in the last 12 months (17.56%) and diabetes (16.59%). An sPESI score ≥ 1 was found in 109 patients (54.77%), indicating an elevated risk for PE-related death in the first 30 days after the acute event (18).

**Table 2:** Socio-demographic and health-related variables of the “‘Lungenembolie Augsburg (LEA)” study sample at baseline.

Characteristics	*n*	Total
Socio-demographic variables		
Age at time of pulmonary embolism [median (Q25, Q75)]	205	63.00 (55.00, 74.00)
Gender	205	
Men		113 (55.12%)
Women		92 (44.88%)
Living alone	201	46 (22.89%)
School education >9 years	204	123 (60.29%)
Health-related variables		
Smoking	205	
Currently		18 (8.78%)
Previously		81 (39.51%)
Never		106 (51.71%)
Prior pulmonary embolism	205	18 (8.78%)
Hypertension	205	102 (49.76%)
Diabetes	205	34 (16.59%)
Prior depression	205	28 (13.66%)
Stroke	205	12 (5.85%)
Cancer in the last 12 months	205	36 (17.56%)
General health status (EQ-5D-5L VAS^a^ score) [median (Q25, Q75)]	140	60.00 (40.00, 75.00)
HADS depression^b^ score [median (Q25, Q75)]	135	5.00 (2.00, 8.00)
Depression^c^		15 (11.11%)
No depression^d^		120 (88.89%)
sPESI score ≥ 1^e^	199	109 (54.77%)

a European quality of life questionnaire—visual analog scale.

b Hospital anxiety and depression scale—subscale depression.

c HADS depression score ≥ 11.

d HADS depression score < 11.

e Simplified pulmonary embolism severity index, which uses age, comorbidities, oxygen saturation, heart rate and blood pressure to predict low (sPESI score < 1) or elevated risk (sPESI score ≥ 1) for 30-day PE-related mortality (18).

Characteristics of the sample at the 3-month follow-up and the 12-month follow-up can be found in Supplementary Tables 1, 2. At the 3-month follow-up, 59 patients (44.70%) had fatigue (FAS score ≥ 24). Compared to patients without fatigue, patients with fatigue had significantly more often prior depression (20.34% vs. 5.48%, *p* = 0.0197) and prior stroke (8.47% vs. 0%, *p* = 0.0162). They also had a significantly lower median EQ-5D-5L VAS score (45.00 vs. 70.00, *p* < 0.0001) and significantly higher median HADS Depression score (8.00 vs. 3.00, *p* < 0.0001) than patients without fatigue.

At the 12-month follow-up, 61 patients (41.22%) had fatigue. These patients were significantly more often female (55.74% vs. 35.63%, *p* = 0.0240) and had significantly more often prior depression (21.31% vs. 8.05%, *p* = 0.0376) than patients without fatigue. Similar to the 3-month follow-up, patients with fatigue at the 12-months had a significantly lower EQ-5D-5L VAS score (50.00 vs. 70.00, *p* = 0.001) and significantly higher median HADS Depression score (8.00 vs. 3.00, *p* = <0.0001) than patients without fatigue.

### 3.2 Characteristics of the comparison samples

Out of the 594 participants included in the MEIA study, 580 completed the FAS and were therefore included in the present analysis. Characteristics of the MEIA analysis sample can be found in Supplementary Table 3. The sample included 251 men (43.28%) and 329 women (56.72%) with a median age of 49.00 years (IQR: 34.00–60.00). The most frequent disease in patients' medical history was COVID-19, with 190 patients having been infected (32.82%). 131 patients (22.63%) reported a diagnosis of hypertension and 46 patients (7.94%) prior depression. The median FAS score was 19.00 (IQR: 15.00–21.00), with 81 patients (13.97%) having a FAS score ≥ 24.

A total of 945 stroke patients participated in the SCHANA study. Of these, 587 patients filled out the FAS at the 3-month follow-up and 533 patients at the 12-month follow-up. For the present analysis we included patients who filled out the FAS during the 3-month follow-up and/or the 12-month follow-up (*n* = 615). Characteristics of the sample can be found in Supplementary Table 4. The sample consisted of 356 men (57.89%) and 259 women (42.11%) with a median age of 70.00 years (IQR: 59.00–78.00). A total of 504 patients (81.95%) had a diagnosis of hypertension, 209 (23.08%) a diagnosis of diabetes and 62 (10.10%) prior depression. The median FAS score at the 3-month follow-up was 21.00 (IQR: 16.33–25.00), with 199 patients (33.90%) having a FAS score ≥ 24. At the 12-month follow-up, the median FAS score was 20.00 (IQR: 16.00–25.00) and 159 patients (29.83%) had a FAS score ≥ 24.

### 3.3 Age- and sex-standardized fatigue prevalence

**Figure 1:**
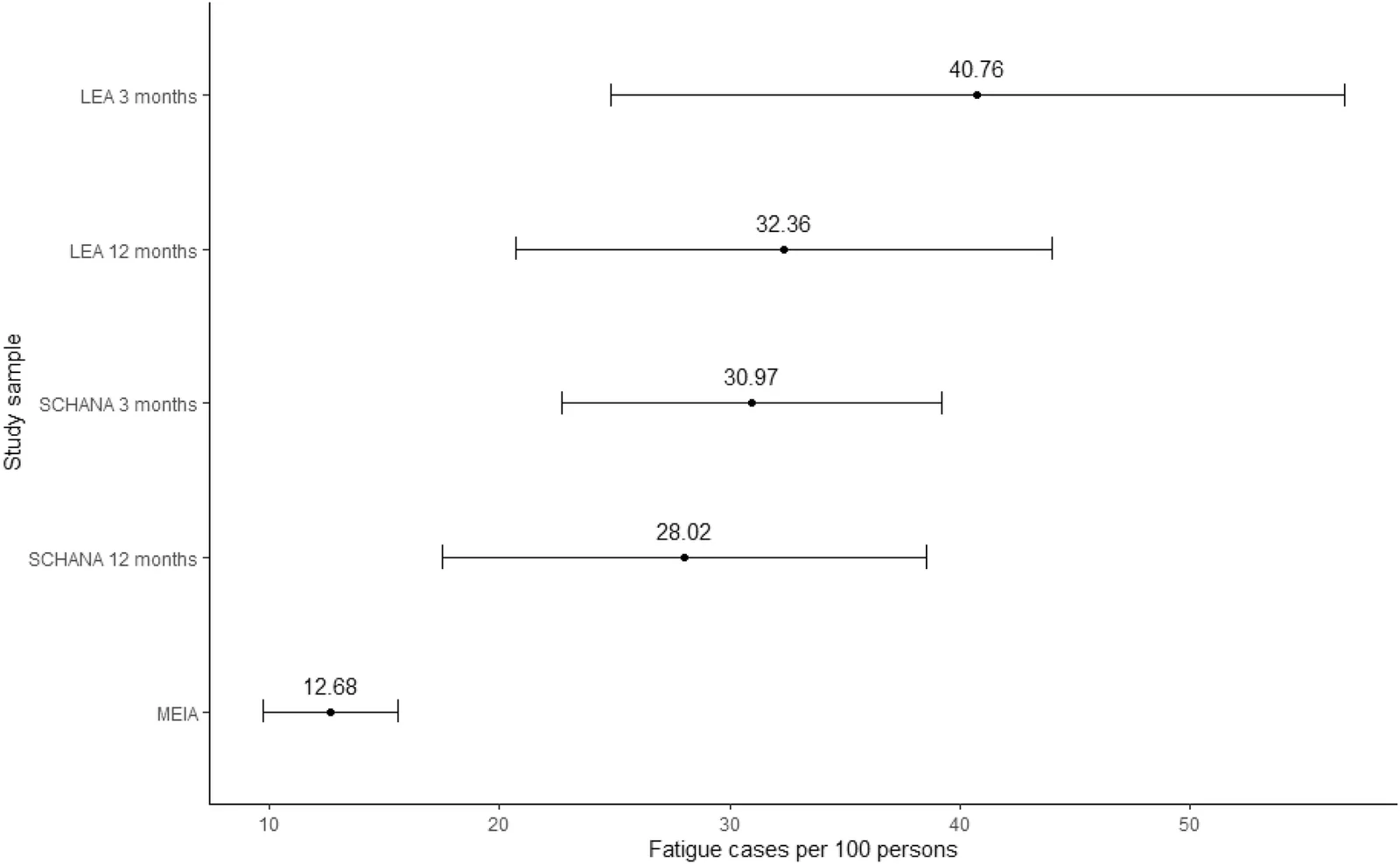
Forest plot of age-sex-standardized numbers of fatigue cases per 100 persons and 95% confidence intervals of the samples. LEA, Lungenembolie Augsburg study; SCHANA, stroke cohort Augsburg; MEIA, metabolism, nutrition and immune system in Augsburg.

Age-sex-standardized fatigue prevalences for the LEA and SCHANA 3-month follow-up and 12-month follow-up samples as well as for the MEIA sample were calculated for the age range of 19–75 + years (see Figure 1). The age-sex-standardized fatigue prevalence at the LEA 3-month follow-up was 40.76% (95% CI: 24.81%–56.71%), while it was 32.36% (95% CI: 20.72%–44.01%) at the LEA 12-month follow-up. At the SCHANA 3-month follow-up, the age-sex-standardized fatigue prevalence was 30.97% (95% CI: 22.73%–39.22%) and 28.02% (95% CI: 17.49%–38.55%) at the 12-month follow-up. In the MEIA sample, the age-sex-standardized fatigue prevalence was 12.68% (95% CI: 9.75%–15.61%). The prevalence for the LEA 3-month follow-up and the SCHANA 3-month follow-up showed no significant difference (*p* = 0.195), neither did the prevalence for the 12-month follow-up of both samples (*p* = 0.195). The fatigue prevalence of the MEIA sample differed significantly from the prevalence at both follow-ups of the LEA sample (3 months *p* < 0.0001; 12 months *p* = 0.0016).

### 3.4 Baseline variables associated with fatigue 3 months after PE

Multiple linear regression models with the FAS score 3 and 12 months after PE as outcome variables are shown in Table 3. Included independent variables were gender, age at time of PE, HADS Depression score, smoking, diabetes, living alone, school education >9 years, sPESI score ≥ 1 and COVID-19. Because we found a significant interaction between gender and HADS Depression score (*β* = −1.01, *p* = 0.0012), models were stratified by gender. For men, higher FAS score was significantly associated with higher baseline HADS Depression score (*β* = 1.92, *p* < 0.0001) as well as school education >9 years (*β* = −3.72, *p* = 0.0491). The model (*p* < 0.0001) explained 74.16% of variance (Adjusted *R*^2^) of the FAS score. In women, HADS Depression score at baseline was significantly associated with the FAS score at the 3-month follow-up (*β* = 0.80, *p* = 0.0057). The model (*p* = 0.0235) explained 27.79% of variance (Adjusted *R*^2^) of the FAS score.

**Table 3:** Multiple linear regression models for the 3-month and the 12-month follow-up of the “‘Lungenembolie Augsburg (LEA)” study with fatigue assessment scale (FAS) score as outcome variable stratified by gender.

Women		3-Month follow-up			12-Month follow-up		
		*n* = 39			*n* = 41		
	Reference	Beta	95% CI	*p*-value	Beta	95% CI	*p*-value
Intercept		30.99	18.40; 43.59	**<0.0001**	28.88	15.99; 41.77	**<0.0001**
Age at time of pulmonary embolism		−0.13	−0.28; 0.02	0.09428	−0.11	−0.26; 0.03	0.1294
HADS depression^a^ score		0.80	0.25; 1.35	**0.0057**	0.55	−0.01; 1.11	0.0545
Smoking (previously)	Smoking (never)	3.99	−1.15; 9.13	0.1231	−0.66	−6.04; 4.72	0.8040
Smoking (currently)	Smoking (never)	−3.08	−12.28; 6.12	0.4987	−3.32	−14.61; 7.97	0.5529
Diabetes (yes)	No	3.77	−2.58; 10.11	0.2343	1.98	−3.68; 7.65	0.4807
Living alone (yes)	No	−2.16	−7.38; 3.05	0.4032	−1.01	−6.13; 4.12	0.6895
School education > 9 years	School education < 10 years	−4.84	−10.21; 0.53	0.0755	−0.10	−5.27; 5.06	0.9683
sPESI^b^ score ≥ 1	sPESI^b^ score < 1	−2.30	−7.01; 2.40	0.3245	−0.59	−5.05; 3.86	0.7883
COVID-19 (yes)	No	−2.10	−6.74; 2.54	0.3616	−0.65	−5.45; 4.15	0.7842

Men		3-month follow-up			12-month follow-up		
		*n* = 45			*n* = 57		
	Reference	Beta	95% CI	*p*-value	Beta	95% CI	*p*-value
Intercept		23.46	11.48; 35.45	**0** **.** **0003**	19.78	5.84; 33.72	**0** **.** **0064**
Age at time of pulmonary embolism		−0.08	−0.23; 0.07	0.3139	−0.04	−0.22; 0.15	0.6702
HADS depression^a^ score		1.92	1.54; 2.30	**<0** **.** **0001**	1.69	1.23; 2.15	**<0** **.** **0001**
Smoking (previously)	Smoking (never)	−2.57	−5.76; 0.61	0.1103	−0.68	−4.29; 2.92	0.7042
Smoking (currently)	Smoking (never)	−1.66	−7.03; 3.72	0.5356	0.11	−6.29; 6.51	0.9729
Diabetes (yes)	No	2.97	−3.67; 9.61	0.3703	−0.57	−7.43; 6.33	0.8735
Living alone	No	−0.69	−5.41; 4.02	0.7669	1.57	−3.59; 6.72	0.5444
School education > 9 years	School education < 10 years	−3.72	−7.42; −0.02	**0** **.** **0491**	−5.28	−8.96; −1.60	**0** **.** **0059**
sPESI^b^ score ≥ 1	sPESI^b^ score < 1	−1.36	−4.70; 1.99	0.4167	−0.26	−3.75; 3.22	0.8796
COVID-19 (yes)	No	−2.68	−5.95; 0.58	0.1045	−1.66	−5.23; 1.92	0.3555

Bolt font indicates statistical significance with *p* < 0.05.

a Hospital anxiety and depression scale (HADS)—subscale depression.

b Simplified pulmonary embolism severity index.

### 3.5 Baseline variables associated with fatigue 12 months after PE

The same variables were included in the multiple linear regression model for the 12-month follow-up (see Table 3). This model also showed a significant interaction between gender and HADS Depression score (*β* = −1.16, *p* = 0.0006). Stratified models showed that in men, HADS Depression score (*β* = 1.69, *p* < 0.0001) and school education >9 years (*β* = −5.28, *p* = 0.0059) were significantly associated with a higher FAS score. The model (*p* < 0.0001) explained 58.77% of variance (Adjusted *R*^2^) of the FAS score. In women, no significant associations between any of the variables and the FAS score 12 months after PE were found. The model for women (*p* = 0.2776) explained 6.28% of variance (Adjusted *R*^2^) of the FAS score.

### 3.6 Sensitivity analysis

Sensitivity analysis of baseline characteristics revealed that patients who only participated in the baseline assessment and did not complete any follow-up were significantly older and had significantly less often school education >9 years than patients included in the analysis sample (see Supplementary Table 5). They were also significantly more often affected by prior diseases such as diabetes, depression, stroke or cancer and by high-risk PE (sPESI score ≥ 1). Furthermore, they had significantly higher HADS Depression scores. Robust multiple linear regression models were compared to the multiple linear regression models to investigate the influence of outliers on the results (see Supplementary Tables 6–9). As results were comparable and estimates for significant predictors remained stable, we assume that our models are robust against outliers. We tested the dependance of the results on the depression score by removing the variable HADS Depression score from the regression models. Without this variable, none of the overall models was statistically significant anymore. The models for men and women at the 3-month follow-up showed no more variables significantly associated with the FAS score (see Supplementary Table 10). In the regression models for men and women at the 12-month follow-up, the variables significantly associated with the FAS score stayed the same as with HADS Depression score included (see Supplementary Table 11).

## 4 Discussion

Our results demonstrate that fatigue is common after PE, with an age-sex-adjusted fatigue prevalence of 40.76% at 3 months and 32.36% at 12 months post-PE. In men, higher baseline depression scores and lower school education levels were significant predictors of fatigue at both time points, while in women, depression at baseline was a significant predictor of fatigue at 3 months after PE.

Studies reporting fatigue prevalence or incidence after PE are rare and their results are difficult to compare with the present study because other databases and fatigue assessments were used. For instance, based on insurance claims, Aggarwal et al. found that 18.5% of patients were newly affected by fatigue and malaise 3–12 months after PE (12). However, fatigue is often initially misdiagnosed by general practitioners, which can lead to under-reporting (36). Therefore, the higher point prevalences based on self-reported questionnaires found in the present study nevertheless seem plausible. The lower fatigue prevalence at 12 months compared with 3 months post-PE found in the present study is consistent with a temporal decline in quality-of-life impairment observed in previous studies (2, 37, 38).

The finding from the present study that fatigue levels in PE patients were significantly higher than those in the general population is not surprising. Although fatigue is a phenomenon also observed in the general population (9, 39), higher fatigue levels have been reported for many other chronic health conditions (34, 40), including stroke (41).

The present study showed that patients after PE and patients after stroke are at least similarly affected by fatigue. In our analysis, the fatigue prevalence in PE patients was higher than in stroke patients at both measurement points, but the differences did not reach statistical significance. Current research shows that the overall prevalence of post-stroke fatigue is around 50%, which is higher than in the SCHANA study (11). This can possibly be explained by the fact that the SCHANA study mainly consisted of stroke patients with mild impairment (14).

In terms of possible predictors of fatigue, a higher depression score has shown to be a key predictor for higher fatigue levels in the present study. In a systematic review, Torossian and Jacelon reported that the positive association between fatigue and depression has been consistently shown in several studies measuring fatigue in chronically ill older individuals (27). A significant positive association between depression and fatigue was also found in stroke patients (14). Fatigue and depression are closely intertwined, as depression can both be predictor of fatigue as well as its consequence, and fatigue can present as a symptom of major depression (27, 28). But they remain distinct diseases, as there are individuals with fatigue who are not depressed and vice versa (42). In our analysis, 80.56% of patients with fatigue 3 months after PE and 76.92% of patients with fatigue 12 months after PE did not have depression. For results to be interpreted accurately, fatigue needs to be assessed independently of depression, and fatigue questionnaires should have no substantial overlap with measures of depressive symptoms (28). This applies to the FAS which was used in the present study (28).

Of interest, in our study gender modified the association between depression and fatigue. The positive association between HADS Depression score and FAS score was more pronounced in men than in women, suggesting that higher depression level at baseline is a stronger predictor for later fatigue in men than in women. Moreover, in men, higher school education level was negatively associated with fatigue. In women, school education level showed no effect. A systematic review described lower school education level being positively associated with fatigue in five out of six reviewed studies on chronically ill patients (27). A German cohort study assessing fatigue in the general population revealed that having a lower school education level is associated with a higher risk for fatigue in men and women (9). When we removed depression as a variable from the regression models for men, lower school education level remained a predictor for fatigue 12 months after PE, but not for fatigue 3 months after PE. The association between lower school educational level and increased risk for depression has been shown in many studies (43). This could indicate a mediating role of depression in the relationship between school education level and fatigue 3 months after PE. Twelve months after PE, school education level predicted fatigue independently of depression and is therefore a better predictor for later fatigue.

Twelve months after PE, women were significantly more likely to experience fatigue than men. Higher fatigue prevalence in women was also reported in the German general population and in many diseases such as heart failure, stroke or COVID-19 (9, 34). The reasons for this gender difference are still unknown. Lim et al. suggest that the higher prevalence for chronic fatigue syndrome in women may be related to gender-specific immune reaction to environmental factors and sex hormones, as well as higher willingness to report symptoms of fatigue in women (44).

PE severity, as assessed by the sPESI, showed no significant association with fatigue. Like in our findings, Valerio et al. found no association between PE risk class at hospitalization and quality of life 3 and 12 months after the acute event (2). They concluded that long-term negative outcomes can occur irrespective of PE severity (2).

The period of data collection covered the COVID-19 pandemic. Fatigue is one of the most prevalent symptoms following the infection with SARS-CoV-2 (33). In our study, having been infected with SARS-CoV-2 was not significantly associated with fatigue after PE. Studies showed that fatigue was not only high in those infected by SARS-CoV-2, but also in the non-infected control groups, which may partly explain our results (9).

In both men and women, a considerable decrease in the explained variance of the FAS score was observed between the measurement points. While fatigue 3 months after PE can be well explained particularly by depression, other unmeasured factors seem to play a greater role in the development of fatigue 12 months after PE.

The regression models for men explained a substantial proportion of the variance in the FAS score at both measurement points (*R*^2^ = 74.16% and 58.77%), while the explained variance was considerably lower in the models for women (*R*^2^ = 27.79% and 6.28%). First, the smaller sample size of the females associated with a loss of statistical power may have contributed to this finding. In addition, it seems possible that unmeasured factors have largely affected the fatigue prevalence in women. Gender differences in terms of coping strategies, stress, sleep and health care utilization may have contributed to these differences and should be considered in future studies (45–48) Furthermore, biological factors such as inflammatory processes as well as sex- and stress hormones may play an important role for the development of fatigue, but have not yet been sufficiently studied (49–52).

Our findings may have practical implications. Since the examined parameters related to physical health were not good indicators of subsequent fatigue, screening patients' mental health at an early stage and therefore facilitating initiation of treatment may be recommended. While the European Society of Cardiology (ESC) guidelines focus on physical consequences of PE and do not include recommendations for psychological care, a position paper to the ESC guidelines suggests considering psychological support for patients with depression 3 months after the acute event (53, 54).

Given the high prevalence of fatigue in our sample, patients who experienced PE should be screened for fatigue during health care visits. The fact that the causes of fatigue are still not fully understood and patients' symptoms are highly heterogenous complicates finding treatment (11). There is no research on treatment of fatigue following PE yet. Initial findings have been obtained for treating post-stroke fatigue, showing that pharmacological treatment, mindfulness-based stress reduction and cognitive training combined with activity training may decrease fatigue scores (55). It is unclear whether these could also be treatment options for fatigue after PE. As fatigue affects many patients after PE, further research is necessary.

Our study has several strengths. First, to our knowledge, this is the first quantitative study investigating the prevalence of fatigue after PE and identifying possible predictors. The longitudinal study design allowed us to determine a temporal relationship between baseline variables and subsequent fatigue. A fatigue questionnaire with high validity and reliability, and without overlapping with symptoms of depression has been used to assess fatigue, enabling a good differentiation between depression and fatigue. Moreover, its applicability for different populations allows a comparison of fatigue levels across these populations and its use facilitates the comparison of study results worldwide. Finally, the two comparative studies were carried out at the same center and the same fatigue questionnaire was used, therefore reducing measurement heterogeneity.

Limitations of our study include the relatively small sample size which may limit the reliability of the results and the generalizability of the results to the overall population of PE patients. Furthermore, it was a single-center study conducted at a university hospital in southern Germany, which could have reduced external validity. The FAS has not been validated in the PE population before, so it remains unknown whether its good psychometric properties shown in stroke patients and the general population could be reproduced (15, 16). Therefore, psychometric validation of the FAS in a PE population should be performed in the future. Fatigue was not assessed immediately after the PE event and therefore we can't rule out that unmeasured baseline fatigue could have acted as a confounder influencing both baseline depression and later fatigue, or as a mediator in the relationship between baseline depression and fatigue 3 months/12 months after PE. Both scenarios would have led to biased results.

Since patients who dropped out after baseline examination had higher depression scores and lower school education levels, we can't rule out attrition bias. Because depression was the key predictor of fatigue in the present study, a drop-out of individuals with high depression scores is likely to lead to an underestimation of fatigue prevalence. Similarly, fatigue prevalence in men may have been underestimated due to the drop-out of individuals with poor school education.

Finally, the samples assessed at 3 months and 12 months consist largely of different participants, which restrict interpretation of changes in fatigue and its predictors over time.

## 5 Conclusions

This is the first quantitative study investigating the prevalence of fatigue after PE and identifying possible predictors. The results show that fatigue is common after PE and should be considered by physicians. Early detection and intervention may help address negative impact on quality of life, health care utilization and incapacity for work. As early depression seems to be a key predictor for later fatigue, we suggest screening patients for depressive symptoms as soon as possible after the acute event and starting psychological treatment if necessary. Besides depression, lower school education level has shown to be a predictor for developing fatigue after PE in men and should be considered when screening patients.

## Data Availability

The datasets presented in this article are not readily available because of data protection aspects but are available in an anonymized form from the Institute of Epidemiology on reasonable request. Requests to access the datasets should be directed to Inge Kirchberger, inge.kirchberger@med.uni-augsburg.de.
