# Supplementary material for: Fatigue after pulmonary embolism—prevalence and predictors
Source: Front Cardiovasc Med. 2026 Aug 24;13:1882131. doi: 10.3389/fcvm.2026.1882131 (PMC13547306; doi:10.3389/fcvm.2026.1882131)
Supplement: Supplementary file 1 [file Datasheet1.pdf]

11 Supplementary Material

Supplementary Table 1 Socio-demographic and health-related variables of the 'Lungenembolie Augsburg (LEA)' sample 3 months after pulmonary embolism stratified by fatigue status

**Socio-demographic variables**	**Total n=132**	**Patients with fatigue[a] n=59 (44.70%)**	**Patients without fatigue[b], n=73 (55.30%)**	**p-value**
Age at time of pulmonary embolism [median (Q_{25}, Q_{75})]	132	67.00 (56.00, 77.00)	64.00 (56.00, 73.00)	0.5348
Gender	132			0.6646
Men		30 (50.85%)	41 (56.16%)	
Women		29 (49.15%)	32 (43.84%)	
Living alone	129	12 (21.43%)	16 (21.92%)	1
School education >9 years	131	29 (50%)	47 (64.38%)	0.1392
**Health-related variables**				
Smoking	132			0.2958
currently		9 (15.25%)	5 (6.85%)	
never		29 (49.15%)	39 (53.42%)	
previously		21 (35.60%)	29 (39.73%)	
Prior pulmonary embolism	132	2 (3.39%)	7 (9.59%)	0.1872
Hypertension	132	34 (57.63%)	31 (42.47%)	0.1194
Diabetes	132	11 (18.64%)	10 (13.70%)	0.594
Prior depression	132	12 (20.34%)	4 (5.48%)	**0.0197**
Stroke	132	5 (8.47%)	0 (0%)	**0.0162**
Cancer (in the last 12 months)	132	15 (25.42%)	10 (13.70%)	0.1373
COVID-19	132	26 (44.07%)	40 (54.80%)	0.2935
General health status (EQ-5D-5L VAS score)[c] [median (Q_{25}, Q_{75})]	92	45.00 (35.00, 60.00)	70.00 (54.50, 80.00)	**<0.0001**
HADS Depression[d] score [median (Q_{25}, Q_{75})]	88	8.00 (4.75, 9.25)	3.00 (1.00, 5.00)	**<0.0001**
Depression [e]		7 (19.44%)	2 (3.85%)	
No depression [f]		29 (80.56%)	50 (96.15%)	
sPESI score ≥ 1[g]	130	35 (61.40%)	38 (52.10%)	0.3746

Bolt font indicates statistical significance with alpha = 0.05

[a] Fatigue Assessment Scale (FAS) score ≥ 24

[b] Fatigue Assessment Scale (FAS) score < 24

[c] European Quality of Life Questionnaire - Visual Analog Scale

[d] Hospital Anxiety and Depression Scale (HADS) - subscale depression

[e] HADS Depression score ≥ 11

[f] HADS Depression score < 11

[g] Simplified Pulmonary Embolism Severity Index, which uses age, comorbidities, oxygen saturation, heart rate and blood pressure to predict low (sPESI score < 1) or elevated risk (sPESI score ≥ 1) for 30-day PE-related mortality (18)

Supplementary Table 2 Socio-demographic and health-related variables of the 'Lungenembolie Augsburg (LEA)' sample 12 months after pulmonary embolism stratified by fatigue status

**Socio-demographic variables**	**Total n= 148**	**Patients with fatigue[a], n=61 (41.22%)**	**Patients without fatigue[b], n=87 (58.78%)**	**p-value**
Age at time of pulmonary embolism [median (Q_{25}, Q_{75})]	148	64.00 (54.00, 76.00)	62.00 (55.00, 69.00)	0.1635
Gender	148			**0.0240**
Men		27 (44.26%)	56 (64.37%)	
Women		34 (55.74%)	31 (35.63%)	
Living alone	147	12 (20.34%)	21 (24.14%)	0.7362
School education >9 years	148	32 (52.46%)	58 (66.67%)	0.116
**Health-related variables**				
Smoking	148			0.6854
currently		4 (6.56%)	7 (8.05%)	
never		35 (57.38%)	43 (49.43%)	
previously		22 (36.07%)	37 (42.53%)	
Prior pulmonary embolism	148	3 (4.92%)	11 (12.64%)	0.1952
Hypertension	148	29 (47.54%)	38 (43.68%)	0.7665
Diabetes	148	14 (22.95%)	9 (10.34%)	0.0639
Prior depression	148	13 (21.31%)	7 (8.05%)	**0.0376**
Stroke	148	5 (8.20%)	2 (2.30%)	0.1249
Cancer (in the last 12 months)	148	10 (16.40%)	12 (13.79%)	0.8391
COVID-19	146	24 (39.34%)	41 (48.23%)	0.3696
General health status (EQ-5D-5L VAS score)[c] [median (Q_{25}, Q_{75})]	107	50.00 (40.00, 65.00)	70.00 (46.25, 80.00)	**0.001**
HADS Depression[d] score [median (Q_{25}, Q_{75})]	102	8.00 (4.00, 10.00)	3.00 (1.50, 6.00)	**<0.0001**
Depression[e]		9 (23.08%)	1 (1.59%)	
No depression [f]		30 (76.92%)	62 (98.41%)	
sPESI score ≥ 1[g]	144	33 (54.10%)	39 (46.99%)	0.4999

Bolt font indicates statistical significance with alpha = 0.05
[a] Fatigue Assessment Scale (FAS) score ≥ 24
[b] Fatigue Assessment Scale (FAS) score < 24
[c] European Quality of Life Questionnaire - Visual Analog Scale
[d] Hospital Anxiety and Depression Scale (HADS) - subscale depression
[e] HADS Depression score ≥ 11
[f] HADS Depression score < 11
[g] Simplified Pulmonary Embolism Severity Index, which uses age, comorbidities, oxygen saturation, heart rate and blood pressure to predict low (sPESI score < 1) or elevated risk (sPESI score ≥ 1) for 30-day PE-related mortality (18)

Supplementary Table 3 Characteristics of the 'Metabolism, Nutrition and Immune System (MEIA)' sample

**Socio-demographic variables**	**Total n=580**	**Total**
Age [median (Q_{25}, Q_{75})]	580	49.00 (34.00, 60.00)
Gender	580	
Men		251 (43.28%)
Women		329 (56.72%)
**Health-related variables**		
Smoking	580	
currently		93 (16.03%)
previously		194 (33.45%)
never		293 (50.52%)
Education	580	
School for ≤ 10 years		20 (3.45%)
School for > 10 years		97 (16.72%)
Apprenticeship		233 (40.17%)
University degree		218 (37.59%)
Doctorate		12 (2.07%)
FAS[a] score [median (Q_{25}, Q_{75})]	580	19.00 (15.00, 21.00)
Fatigue[b]		81 (13.97%)
No fatigue[c]		499 (86.03%)
Hypertension	579	131 (22.63%)
Depression	579	46 (7.94%)
Diabetes	579	22 (3.80%)
Heart failure	579	9 (1.55%)
Coronary heart disease	579	24 (4.15%)
Stroke	579	12 (2.07%)
Cancer	580	43 (7.41%)
COVID-19	579	190 (32.82%)

[a] Fatigue Assessment Scale (FAS)
[b] FAS score ≥ 24
[c] FAS score < 24

Supplementary Table 4 Characteristics of the 'Stroke Cohort Augsburg (SCHANA)' sample

**Socio-demographic variables**	**Total n=615**	**Total**
Age [median (Q_{25}, Q_{75})]	615	70.00 (59.00, 78.00)
Gender	615	
Men		356 (57.89%)
Women		259 (42.11%)
School education > 9 years	591	249 (42.13%)
**Health-related variables**		
Smoking	615	
currently		89 (14.47%)
previously		260 (42.28%)
never		266 (43.25%)
Hypertension	615	504 (81.95%)
Coronary heart disease	614	169 (17.43%)
Prior depression	614	62 (10.10%)
Cancer	614	90 (14.66%)
Diabetes	611	209 (23.08%)
PHQ-9[a] score (after 3 months) [median (Q_{25}, Q_{75})]	569	0.00 (0.00, 2.00)
Depression[b]		20 (3.51%)
No depression[c]		549 (96.49%)
PHQ-9[a] score (after 12 months) [median (Q_{25}, Q_{75})]	520	0.00 (0.00, 2.00)
Depression[b]		13 (2.50%)
No depression[c]		507 (97.50%)
FAS[d] score (after 3 months) [median (Q_{25}, Q_{75})]	587	21.00 (16.33, 25.00)
Fatigue [e]		199 (33.90%)
No fatigue[f]		388 (66.10%)
FAS[d] score (after 12 months) [median (Q_{25}, Q_{75})]	533	20.00 (16.00, 25.00)
Fatigue [e]		159 (29.83%)
No fatigue[f]		374 (70.17%)

[a] Patient Health Questionnaire (PHQ-9)
[b] PHQ-9 score ≥ 10
[c] PHQ-9 score < 10
[d] Fatigue Assessment Scale (FAS)
[e] FAS score ≥ 24
[f] FAS score < 24

Supplementary Table 5 Comparison of characteristics of patients in the 'Lungenembolie Augsburg (LEA)' analysis sample and patients who dropped out after baseline assessment of the 'Lungenembolie Augsburg (LEA)' study

**Socio-demographic variables**	**n**	**Patients in the analysis sample**	**n**	**Patients who dropped out after baseline**	**p-value**
Age at time of pulmonary embolism [median (Q_{25}, Q_{75})]	205	63.00 (55.00, 74.00)	242	69.00 (56.00, 79.75)	**0.0053**
Gender	205		242		0.0771
Men		113 (55.12%)		112 (46.28%)	
Women		92 (44.88%)		130 (53.72%)	
Living alone	201	46 (22.89%)	227	72 (31.72%)	0.0533
School education > 9 years	204	123 (60.29%)	239	100 (41.84%)	**0.0008**
**Health-related variables**					
Smoking	205		238		0.2826
Currently		18 (8.78%)		32 (13.45%)	
Previously		81 (39.51%)		93 (39.08%)	
Never		106 (51.71%)		113 (47.48%)	
Prior pulmonary embolism	205	18 (8.78%)	239	31 (12.97%)	0.2103
Hypertension	205	102 (49.76%)	239	136 (56.90%)	0.1585
Diabetes	205	34 (16.59%)	239	60 (25.10%)	**0.0381**
Prior depression	205	28 (13.66%)	238	54 (22.69%)	**0.0205**
Stroke	205	12 (5.85%)	239	32 (13.39%)	**0.0128**
Cancer (in the last 12 months)	205	36 (17.56%)	239	74 (30.96%)	**0.0016**
EQ-5D-5L VAS[a] score [median (Q25, Q75)]	140	60.00 (40.00, 75.00)	92	50.00 (38.75, 70.50)	0.0617
HADS Depression[b] score [median (Q25, Q75)]	135	5.00 (2.00, 8.00)	88	6.00 (4.00, 9.00)	**0.0044**
Depression[c]		15 (11.11%)		18 (20.45%)	
No depression[d]		120 (88.89%)		70 (79.55%)	
sPESI score ≥ 1[e]	199	109 (54.77%)	221	170 (76.92%)	**< 0.0001**

Bolt font indicates statistical significance with alpha = 0.05

[a] European Quality of Life Questionnaire - Visual Analog Scale

[b] Hospital Anxiety and Depression Scale (HADS) - subscale depression

[c] HADS Depression score ≥ 11

[d] HADS Depression score < 11

[e] Simplified Pulmonary Embolism Severity Index, which uses age, comorbidities, oxygen saturation, heart rate and blood pressure to predict low (sPESI score < 1) or elevated risk (sPESI score ≥ 1) for 30-day PE-related mortality (18)

Supplementary Table 6 Comparison of multiple linear regression model and robust multiple linear regression model for men at the 3-month follow-up with Fatigue-Assessment-Scale (FAS) score as the outcome variable

		Linear Model		***Robust Linear Model***
Variable	**Reference**	**Beta**	**p-value**	**Beta**
Intercept		23.46	**0.0003**	22.35
Age at time of pulmonary embolism		-0.08	0.3139	-0.07
HADS Depression[a] score		1.92	**< 0.0001**	1.89
Smoking (previously)	Smoking (never)	-2.57	0.1103	-2.46
Smoking (currently)	Smoking (never)	-1.66	0.5356	-1.28
Diabetes (yes)	No	2.97	0.3703	2.89
Living alone (yes)	No	-0.69	0.7669	-1.03
School education > 9 years	School education < 10 years	-3.72	**0.0490**	-2.97
sPESI score ≥ 1[b]	sPESI score < 1	-1.36	0.4167	-0.81
COVID-19 (yes)	No	-2.68	0.1045	-2.01

Bolt font indicates statistical significance with alpha = 0.05
[a] Hospital Anxiety and Depression Scale (HADS) – subscale depression
[b] Simplified Pulmonary Embolism Severity Index, which uses age, comorbidities, oxygen saturation, heart rate and blood pressure to predict low (sPESI score < 1) or elevated risk (sPESI score ≥ 1) for 30-day PE-related mortality (18)

Supplementary Table 7 Comparison of multiple linear regression model and robust multiple linear regression model for men at the 12-month follow-up with Fatigue-Assessment-Scale (FAS) score as the outcome variable

		Linear Model		***Robust Linear Model***
Variable	**Reference**	**Beta**	**p-value**	**Beta**
Intercept		19.78	**0.0064**	20.55
Age at time of pulmonary embolism		-0.04	0.6702	-0.05
HADS Depression[a] score		1.69	**< 0.0001**	1.61
Smoking (previously)	Smoking (never)	-0.68	0.7042	-0.67
Smoking (currently)	Smoking (never)	0.11	0.9729	0.97
Diabetes (yes)	No	-0.57	0.8735	0.13
Living alone (yes)	No	1.57	0.5444	1.56
School education > 9 years	School education < 10 years	-5.28	**0.0059**	-4.97
sPESI score ≥ 1[b]	sPESI score < 1	-0.26	0.8796	0.24
COVID-19 (yes)	No	-1.66	0.3555	-2.57

Bolt font indicates statistical significance with alpha = 0.05
[a] Hospital Anxiety and Depression Scale (HADS) – subscale depression
[b] Simplified Pulmonary Embolism Severity Index, which uses age, comorbidities, oxygen saturation, heart rate and blood pressure to predict low (sPESI score < 1) or elevated risk (sPESI score ≥ 1) for 30-day PE-related mortality (18)

Supplementary Table 8 Comparison of multiple linear regression model and robust multiple linear regression model for women at the 3-month follow-up with Fatigue-Assessment-Scale (FAS) score as the outcome variable

		Linear Model		***Robust Linear Model***
Variable	**Reference**	**Beta**	**p-value**	**Beta**
Intercept		30.99	**< 0.0001**	29.96
Age at time of pulmonary embolism		-0.13	0.0943	-0.11
HADS Depression[a] score		0.80	**0.0059**	0.81
Smoking (previously)	Smoking (never)	3.99	0.1231	4.50
Smoking (currently)	Smoking (never)	-3.08	0.4987	-0.98
Diabetes (yes)	No	3.77	0.2343	4.64
Living alone (yes)	No	-2.16	0.4032	-2.51
School education > 9 years	School education < 10 years	-4.84	0.0755	-4.74
sPESI score ≥ 1[b]	sPESI score < 1	-2.30	0.3245	-2.68
COVID-19 (yes)	No	-2.10	0.3616	-3.00

Bolt font indicates statistical significance with alpha = 0.05
[a] Hospital Anxiety and Depression Scale (HADS) – subscale depression
[b] Simplified Pulmonary Embolism Severity Index, which uses age, comorbidities, oxygen saturation, heart rate and blood pressure to predict low (sPESI score < 1) or elevated risk (sPESI score ≥ 1) for 30-day PE-related mortality (18)

Supplementary Table 9 Comparison of multiple linear regression model and robust multiple linear regression model for women at the 12-month follow-up with Fatigue-Assessment-Scale (FAS) score as the outcome variable

		Linear Model		***Robust Linear Model***
Variable	**Reference**	**Beta**	**p-value**	**Beta**
Intercept		28.88	**< 0.0001**	27.41
Age at time of pulmonary embolism		-0.11	0.1294	-0.09
HADS Depression[a] score		0.55	0.0545	0.60
Smoking (previously)	Smoking (never)	-0.66	0.8040	-0.29
Smoking (currently)	Smoking (never)	-3.32	0.5529	-2.39
Diabetes (yes)	No	1.98	0.4807	2.46
Living alone (yes)	No	-1.01	0.6895	-1.19
School education > 9 years	School education < 10 years	-0.10	0.9683	-0.38
sPESI[b] score ≥ 1	sPESI[b] score < 1	-0.59	0.7883	-0.97
COVID-19 (yes)	No	-0.65	0.7942	-1.53

Bolt font indicates statistical significance with alpha = 0.05
[a] Hospital Anxiety and Depression Scale (HADS) – subscale depression
[b] Simplified Pulmonary Embolism Severity Index, which uses age, comorbidities, oxygen saturation, heart rate and blood pressure to predict low (sPESI score < 1) or elevated risk (sPESI score ≥ 1) for 30-day PE-related mortality (18)

Supplementary Table 10 Final multiple linear regression model for the 3-month follow-up without Hospital Anxiety and Depression Scale (HADS) depression score as a variable

		Men			***Women***		
	**Reference**	**Beta**	**95% CI**	**p-value**	**Beta**	**95% CI**	**p-value**
Intercept		30.72	14.66; 46.79	**0.0003**	32.70	21.84; 43.56	**<0.0001**
Age at time of pulmonary embolim		-0.11	-0.31; 0.10	0.3009	-0.11	-0.26; 0.04	0.158
Smoking (previously)	Smoking (never)	-1.62	-6.31; 3.07	0.4932	1.80	-2.61; 6.21	0.416
Smoking (currently)	Smoking (never)	-0.14	-7.31; 7.04	0.9699	0.74	-7.72; 9.20	0.862
Diabetes (yes)	No	1.90	-5.45; 9.25	0.6076	0.66	-5.43; 6.74	0.829
Living alone (yes)	No	6.29	-1.16; 13.73	0.0964	-0.91	-5.68; 3.86	0.703
School education > 9 years	School education < 10 years	-2.17	-7.27; 2.94	0.3989	-2.13	-6.49; 2.23	0.332
sPESI[a] score ≥ 1	sPESI[a] score < 1	2.74	-1.91; 7.41	0.2430	-0.62	-5.08; 3.85	0.782
COVID-19 (yes)	No	-3.78	-8.38; 0.82	0.1054	-3.16	-7.41; 1.10	0.142

Bolt font indicates statistical significance with alpha = 0.05

[a] Simplified Pulmonary Embolism Severity Index, which uses age, comorbidities, oxygen saturation, heart rate and blood pressure to predict low (sPESI score < 1) or elevated risk (sPESI score ≥ 1) for 30-day PE-related mortality (18)

Supplementary Table 11 Final multiple linear regression model for the 12-month follow-up without Hospital Anxiety and Depression Scale (HADS) depression score as a variable

		Men			***Women***		
	**Reference**	**Beta**	**95% CI**	**p-value**	**Beta**	**95% CI**	**p-value**
Intercept		23.87	10.53; 37.20	**0.0006**	32.05	20.91; 43.19	**<0.0001**
Age at time of pulmonary embolism		0.03	-0.16; 0.21	0.7797	-0.11	-0.25; 0.04	0.146
Smoking (previously)	Smoking (never)	-0.73	-4.85; 3.39	0.7255	0.24	-4.92; 5.39	0.927
Smoking (currently)	Smoking (never)	3.96	-3.40; 11.31	0.2876	-1.50	-11.53; 8.52	0.765
Diabetes (yes)	No	1.24	-4.89; 7.36	0.6882	3.14	-2.39; 8.67	0.260
Living alone (yes)	No	-0.47	-6.45; 5.50	0.8758	-2.01	-6.94; 2.93	0.418
School education > 9 years	School education < 10 years	-5.53	-9.59; -1.46	**0.0084**	1.14	-3.47; 5.75	0.622
sPESI score[a] ≥ 1	sPESI[a] score < 1	1.47	-2.48; 5.42	0.4608	-0.46	-4.77; 3.86	0.832
COVID-19 (yes)	No	-2.51	-6.53; 1.51	0.2176	-0.93	-5.57; 3.70	0.687

Bolt font indicates statistical significance with alpha = 0.05

[a] Simplified Pulmonary Embolism Severity Index, which uses age, comorbidities, oxygen saturation, heart rate and blood pressure to predict low (sPESI score < 1) or elevated risk (sPESI score ≥ 1) for 30-day PE-related mortality (18)
